# Gene novelty and gene family expansion in the early evolution of Lepidoptera

**DOI:** 10.1186/s12864-025-11338-x

**Published:** 2025-02-19

**Authors:** Asia E. Hoile, Peter W. H. Holland, Peter O. Mulhair

**Affiliations:** Department of Biology, University of Oxford, Mansfield Road, Oxford, OX1 3SZ UK

**Keywords:** Insect evolution, HGT, Gene duplication, Genome evolution

## Abstract

**Background:**

Almost 10% of all known animal species belong to Lepidoptera: moths and butterflies. To understand how this incredible diversity evolved we assess the role of gene gain in driving early lepidopteran evolution. Here, we compared the complete genomes of 115 insect species, including 99 Lepidoptera, to search for novel genes coincident with the emergence of Lepidoptera.

**Results:**

We find 217 orthogroups or gene families which emerged on the branch leading to Lepidoptera; of these 177 likely arose by gene duplication followed by extensive sequence divergence, 2 are candidates for origin by horizontal gene transfer, and 38 have no known homology outside of Lepidoptera and possibly arose via de novo gene genesis. We focus on two new gene families that are conserved across all lepidopteran species and underwent extensive duplication, suggesting important roles in lepidopteran biology. One encodes a family of sugar and ion transporter molecules, potentially involved in the evolution of diverse feeding behaviours in early Lepidoptera. The second encodes a family of unusual propeller-shaped proteins that likely originated by horizontal gene transfer from *Spiroplasma* bacteria; we name these the Lepidoptera *propellin* genes.

**Conclusion:**

We provide the first insights into the role of genetic novelty in the early evolution of Lepidoptera. This gives new insight into the rate of gene gain during the evolution of the order as well as providing context on the likely mechanisms of origin. We describe examples of new genes which were retained and duplicated further in all lepidopteran species, suggesting their importance in Lepidoptera evolution.

**Supplementary Information:**

The online version contains supplementary material available at 10.1186/s12864-025-11338-x.

## Background

Diversification and adaptation depend on genetic change but associating genomic drivers underpinning phenotypic change is challenging. Many studies have approached this problem by starting with phenotypic polymorphisms within a species or differences between closely related species and then using genomic and experimental approaches to identify underlying causative mutations. Several of these studies have uncovered sequence changes in non-coding DNA affecting the expression of conserved genes [1–4]. Other studies have identified coding sequence changes causing amino acid substitutions, or loss of function, as causative mutations that were subsequently fixed under selection [5–7]. It is clear, however, that changes in existing genes, whether they affect gene expression or protein sequence, cannot explain all adaptive evolution. Perhaps the best evidence lies in comparative genomics: when genome sequences are compared ample evidence is uncovered for the role of gene number variation, gene duplications, and gene novelty in driving evolution and adaptation [8–13].

Gene novelty is a multi-faceted concept [14–17]. We define novel genes as protein-coding loci that are lineage-specific (i.e. taxonomically restricted genes), without close homologues in other taxa [18]. This is a pragmatic definition rather than a mechanistic one since we cannot always determine the mechanism by which a novel gene arose. The mode of origin of taxonomically restricted genes might be gene duplication followed by extensive sequence divergence [19–24], fusion of distinct loci or a transposable element into a pre-existing locus [25–30], horizontal gene transfer [31–33] or de novo origin from non-coding DNA [17, 34–36]. Whatever the mode of origin, novel genes likely reflect novel biology as they will encode proteins with potentially distinct activity or function not present in the outgroup taxa. Examples in arthropods include horizontally acquired genes from bacteria underpinning adaptations to phytophagy [37] or male courtship behaviour in moths and butterflies [32], and divergent gene duplicates recruited for limb patterning in water striders [38].

Here we investigate the origin of novel genes in the early evolution of the insect order Lepidoptera. Lepidoptera are a holometabolous order of insects consisting of the moths and butterflies and comprise nearly 160,000 described species or 8–10% of known animal species on the planet [39]. The oldest members of the Lepidoptera crown group are estimated to have appeared in the Late Carboniferous (~ 300 mya) and were likely pollen feeders, with the evolution of a tube-like proboscis and nectar feeding occurring later in the Middle Triassic (∼240 Ma). Today the Lepidoptera inhabit almost all terrestrial ecosystems, displaying a large variety of ecological adaptations relating to feeding, defence, and survival [39–41]. Larvae of the earliest lineages were likely endophagous, feeding internally in the tissue of nonvascular land plants, with adults possessing mandibulate chewing mouthparts (as seen in extant members of the family Micropterigidae) suitable for pollen feeding [42, 43]. A period of diversification early in the evolution of Lepidoptera coincided with the development of the tube-like proboscis, used by adults to feed on nectar, and the expansion of angiosperms. The remarkable diversity present in Lepidoptera today can be attributed to continued co-evolution with diverse angiosperm lineages, major transitions in morphology and habitat, and the emergence of diverse feeding behaviours [41].

To assess whether novel genes arose in the early evolution of Lepidoptera, and whether any of these underwent further gene family expansion, we require complete genome sequences from a dense sampling of Lepidoptera and related insect orders. Previous studies have constructed deep-level phylogenies of Lepidoptera using a large density of species but relatively few loci [39], while other studies have studied specific gene families in depth [44–46]. Large genomic datasets have only recently become available through sequencing consortia such as the Darwin Tree of Life Project [47] affiliated to the Earth Biogenome Project [48]. Here, we avail of this data by analysing 115 high quality insect genomes and identify 217 novel genes that arose on the stem lineage of Lepidoptera and 541 novel genes that arose on the stem lineage of the Ditrysia, a major clade encompassing most of lepidopteran diversity [49]. We infer the likely modes of origin for these novel genes. We then focus attention on two gene families gained on the ancestral lepidopteran branch that were subsequently retained across all species, suggestive of recruitment to important roles in lepidopteran biology. One is a gene family encoding divergent sugar transporter proteins; the other is a likely horizontal gene transfer from bacteria.

## Materials and methods

### Gene family construction and discovery of novel genes

Proteome data from 99 species of Lepidoptera and 16 other arthropod species (Supplementary Table S1) were obtained from Ensembl Rapid Release (rapid.ensembl.org; accessed February 2023); taxon sampling was based on obtaining robust phylogenetic coverage across Lepidoptera while also preferentially selecting species with proteome predictions based on the Ensembl genebuild annotation pipeline (i.e. annotation which incorporated RNA sequence data). Primary transcripts were obtained from the predicted proteome data and Orthofinder v2.3.14 was run with default parameters to determine orthogroups within the dataset [50]. To relate these to a species tree, amino acid sequences from 25 single copy orthologues present in all species, as obtained from the Orthofinder output, were aligned using MAFFT v7.505 [51], trimmed using trimAl v1.4.rev15 build [52], and concatenated with PhyKIT [53]. This concatenated alignment was used to generate a species tree using IQ-TREE version 2.0-rc1 with 1000 bootstrap iterations, the given model LG + G4 and option -nt AUTO which automatically determines the best number of cores given the current data and computer capacity [54]. Orthogroups gained at nodes of interest (i.e. the branch leading to Lepidoptera and the branch leading to Ditrysia) were extracted using Orthoparser (github.com/PeterMulhair/ortho_parser). To test further whether orthogroups inferred by the analysis to be specific to Lepidoptera were actually present in outgroups but missing from predicted proteomes, Trichoptera genomes annotated by the alternative Augustus-Gaius pipeline (BRAKER) [55] were analysed. This was carried out using a BLASTp search of the orthogroups against the trichopteran BRAKER proteomes to find any potential missing homologues (using an e-value cutoff of 1e-5 and filtering hits above 25% sequence identity match along with query and subject coverage of 60% to remove hits due to partial homology). Downstream of these steps, genes within orthogroups were analysed by exploring gene copy number, conducting synteny analyses, and generating expression matrices using publicly available RNAseq data. Figures including phylogenetic trees and heatmaps were generated in R using ggtree v3.6.2 [56], ggplot2 v3.4.4 [57], and Pheatmap v1.0.12. Protein models were predicted using AlphaFold (ColabFold v1.5.5: AlphaFold2) [58] and imported into Chimera v1.18 [59]. Molecular graphics and analyses of protein models were performed with UCSF Chimera, developed by the Resource for Biocomputing, Visualization, and Informatics at the University of California, San Francisco, with support from NIH P41-GM103311. Chromosome plots showing gene positions were created using RIdeogram v0.2.2 [60].

### Phylogenetic analysis of gene families

Phylogenetic trees of the two gene families of interest were built by aligning deduced protein sequences using MAFFT v7.505 followed by trimming using trimAl v1.4.rev15 build and tree building using maximum likelihood in IQ-TREE version 2.0-rc1. Trees were visualised using ggtree v3.6.2 in Rstudio. In the sugar transporter orthogroup analyses, PfamScan (command line tool pfam_scan.pl) was used to search each orthogroup against the Pfam-A.hmm database with cutoff –cut_ga and an e-value threshold of 1e-3 [61] to annotate functional domains in each gene. This was used to detect additional gene families labelled as belonging to sugar transporters (possessing Pfam domain Sugar_tr; PF00083), followed by phylogenetic analysis including *Drosophila* and other arthropod SLC sequences to infer the class of SLC each orthogroup belonged to [62]. In the propeller protein analyses, putative HGT was investigated using a BLASTp search (e-value threshold of 1e-3) [63] against the BLAST nr database with all lepidopteran sequences removed (Supplementary Table S3). The source of the HGT was then inferred by building a gene tree from the BLAST hits. Additional orthogroups in our datasets possessing the *propellin* gene were discovered by running a BLASTp search of the initial orthogroup (OG0000175) against all orthogroups in our dataset, retaining only those with percent identity equal to or above 25% and query and subject equal to or above 60%. This uncovered 8 additional homologous orthogroups, each of which contained only lepidopteran species. To further test the likely mode of origin of each of the 9 orthogroups, we carried out sequence similarity searches against the non-redundant protein sequence database (nr) and the core nucleotide database (core_nt) using a set of 10 representative species from each of the orthogroups (Supplementary Table S4). In one of the orthogroups (OG0008135), two of the species had hits against genes/proteins belonging to other insects. To test whether these BLAST hits represented true homologs, or the result of spurious homology, we aligned both insect and *Spiroplasma* proteins to a *Manduca sexta* propellin protein. This was carried out using the RCSB pairwise structure alignment tool [64].

### Gene expression quantification

RNA-seq data for *Bombyx mori* were obtained from NCBI datasets PRJDB8614 and PRJNA675719 [65, 66], for *Danaus plexippus* from PRJNA663267 [67], and for *Papilio machaon* from PRJNA270386 [68]. RNA reads were trimmed using Trimmomatic v0.39 [69], and mapped to the reference genome using STAR 2.7.10b [70]. Stringtie v2.2.1 was used to quantify expression in each of the species datasets [71] and expression matrices were generated in RStudio using Pheatmap. Where multiple samples were available for a given tissue of lifestage, these were averaged to give one value.

### Gene synteny analysis

Synteny analyses were used to test orthology of genes within and beyond Lepidoptera. For genes of interest, the gene ID, chromosome number, and location were determined from the genome annotation and gene track browser on Ensembl Rapid Release [72]. Two conserved ‘marker genes’ either side of the gene of interest were chosen and BLASTp searches (using Ensembl default parameters) conducted against the genomes of four Lepidoptera (*Danaus plexippus*, *Papilio machaon*, *Tinea trinotell*a and *Micropterix aruncella*) and eight outgroups (*Limnephilus lunatus*, *Limnephilus marmoratus*, *Limnephilus rhombicus*, *Glyphotaelius pellucidus*, *Bibio marci*, *Drosophila melanogaster*, *Adalia bipunctata* and *Vespula vulgaris*). These data were used to compare chromosomal organisation and gene neighbourhoods surrounding the genes of interest, revealing if individual genes within lepidopteran orthology groups were 1:1 homologues between species and also whether highly divergent orthologues were present in outgroups.

## Results

### Novel genes emerging at the base of Lepidoptera

**Fig. 1:**
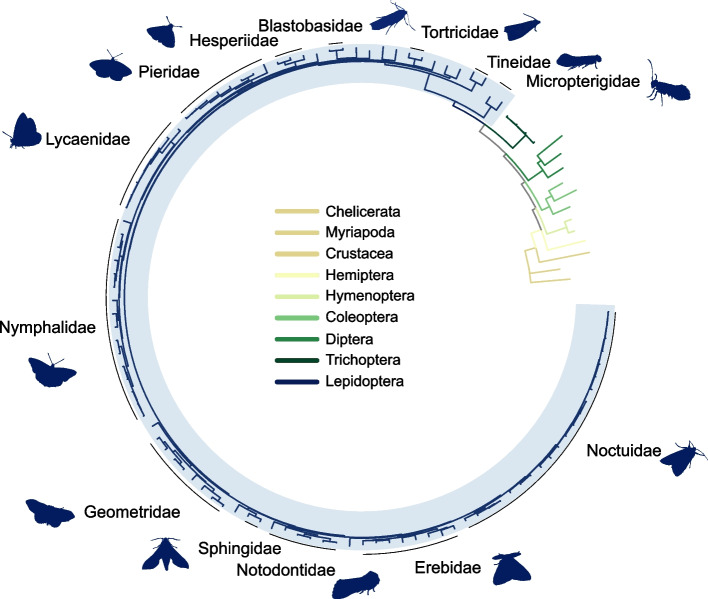
Molecular phylogenetic tree of the 99 lepidopteran species from 24 families and 16 outgroup species inferred from 25 single-copy orthologues. Branches are coloured by insect order; species belonging to the named lepidopteran families are labelled with black lines on the outside of the tree

To build a framework for comparative analyses, a phylogenetic tree was built from 25 single copy genes from 115 species, comprising 99 Lepidoptera species representing 24 families, and 16 outgroup taxa (Fig. 1, Supplementary Table S1). The tree is broadly consistent with previously hypothesized evolutionary relationships, including placing the Micropterigidae family (*Micropterix aruncella* and *Neomicropterix facetella* in our dataset) sister to the rest of the lepidopteran lineages, the presence of the large, established groups of Ditrysia, Apoditrysia, and Macroheterocera [39], and recovering monophyletic groups for all taxonomic families in the dataset [39, 49] (Fig. 1).

**Fig. 2:**
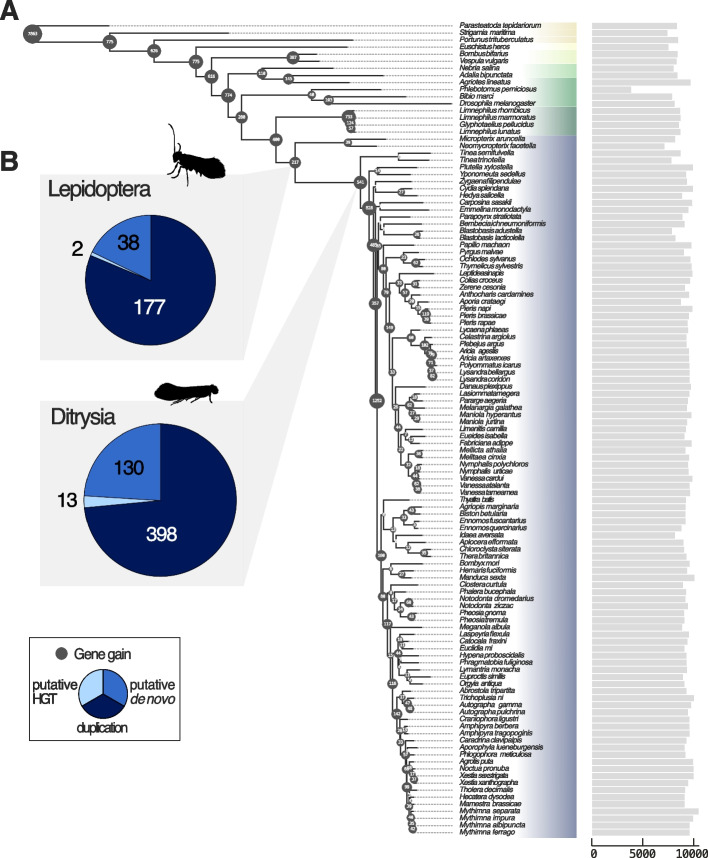
**A** Species tree showing numbers of orthogroups gained at each phylogenetic node. Insect orders are separated by colours. Bar chart to the right of the tree displays the total number of orthogroups identified in each species. **B** Pie charts show the number of orthogroups originating at the Lepidoptera and Ditrysia nodes and proportions of the putative modes of new gene origin

To identify novel genes or novel gene families that emerged early in lepidopteran evolution, we first constructed homologous gene groups (‘orthogroups’) using OrthoFinder [50]. Novel gene families here are defined as orthogroups present in a clade but missing from all outgroup taxa i.e. taxonomically restricted genes. We filtered the complete set of orthogroups to only retain those present in greater than two species. To place each of these orthogroups onto the species tree, we took the parsimonious assumption that the common ancestor of all species present in each orthogroup represented the node of origin (Fig. 2A). We identified 217 putative novel gene families originating on the branch leading to Lepidoptera (Fig. 2A).

To assess the mode of origin for each gene family we applied Pfam annotations to search for protein domains (indicative of duplication and divergence from pre-existing genes) as well as carrying out sequence similarity searches against metazoan (excluding Lepidoptera; further suggestive of duplication) and non-metazoan sequences (suggestive of HGT) from the nr protein database. We deduce that the majority of novel gene families (177 orthogroups) which originated along the lepidopteran branch likely arose via duplication followed by extensive sequence divergence (Fig. 2B). Putative HGTs accounted for only two orthogroups, as indicated by presence in Lepidoptera and non-metazoan proteomes but absent from animals other than Lepidoptera. We suggest that 38 orthogroups are potential orphan genes, candidates for origin by de novo gene genesis, although further analysis and additional data would be needed to test this hypothesis. We also detected 541 putative novel orthogroups on the branch leading to Ditrysia (representing all species outside of Micropterigidae in our dataset) (Fig. 2B). Of these, 398 likely arose from duplication, 13 via HGT, and 130 genes potentially originated de novo.

**Fig. 3:**
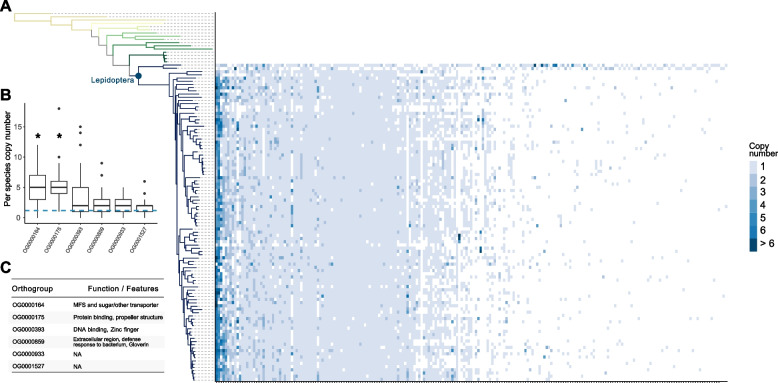
Copy number of genes gained on the ancestral node of Lepidoptera. **A** Heatmap (right) showing gene copy number for each orthogroup originating at the Lepidoptera node mapped to the species tree (left). Lepidoptera node is labelled with a blue circle. Orthogroups on the right-hand side of the figure have genes present in few species and may include spurious homologies. **B** Boxplots showing copy number variation per species in the top 6 orthogroups present in all or most lepidopteran species. Blue broken line signifies the mean copy number per species for all orthogroups. Orthogroups OG0000164 and OG0000175 have a mean copy number significantly different from the mean copy number of lepidopteran orthogroups, as signified by an asterisk (*p* < 0.05). **C** Table showing functions and features from six orthogroups deviating above the average copy number per orthogroup

We hypothesized that novel genes of particular importance to lepidopteran biology would be present in most species of Lepidoptera analysed, with little or no gene loss after gene emergence. Furthermore, some genes of functional importance may have undergone duplication and divergence since their emergence [73]. We therefore calculated gene copy number for every orthogroup originating at the Lepidoptera node and plotted these as a heatmap against a phylogenetic tree (Fig. 3A). Approximately half of the 217 orthogroups showed a scattered phylogenetic distribution (present in a low number of species within Lepidoptera); these may represent genes that are frequently lost, or which underwent extensive sequence divergence within Lepidoptera complicating orthology assignment (right-hand columns in Fig. 3A). 87 orthogroups are present in 75% or more of the lepidopteran species in this dataset, with sporadic gene loss and occasional gene duplication on some internal branches (left-hand columns in Fig. 3A). To identify orthogroups with higher rates of duplication patterns, we first determined that the data does not follow a normal distribution (positive, non-symmetric, right skew) and is non-parametric (Anderson–Darling test, *p* < 0.05), and that at least one orthogroup has a gene copy distribution across species which differs from the mean number of gene copies per orthogroup per species (Kruskal–Wallis rank test, *p* < 0.05; mean number of gene copies = 1.1645). We found that two orthogroups deviate significantly from the mean number of gene copies within a given orthogroup gained at the lepidopteran node: OG0000164 and OG0000175 (Dunn test, *p* < 0.0001; Fig. 3B). These two orthogroups have the highest variation in copy number, implying they have undergone extensive gene duplication within Lepidoptera, and they are also present in every lepidopteran species analysed. Sequence homology from BLASTp searches and domain annotation from Pfam revealed that these proteins have a putative sugar transporter domain (OG0000164; MFS and Sugar/other transporter, PF00083.27, GO:0016020|GO:0022857|GO:0055085) and a 6-bladed beta propeller 3D structure (OG0000175; GO:0005515) (Fig. 3C). To determine whether there were any functions enriched in the full set of 217 orthogroups gained on the lepidopteran node, we analysed the functional domains of each to determine whether there were any categories which were significantly overrepresented. Although no functional categories were found to be enriched within this dataset, approximately 9% of the orthogroups (19 out of 217) were found to contain a zinc finger domain (Supplementary Table S2). We also discover that the Gloverin gene family (OG0000859) emerged on the branch leading to Lepidoptera (Fig. 3C). The *gloverin* gene has previously been described as a lepidopteran novelty, and we confirm its emergence coincident with the evolution of Lepidoptera, where it has been retained in 86 of the 99 lepidopteran species in our dataset including *Micropterix aruncella* (Fig. 3A). Gloverin, first purified from *Hyalophora gloveri* [74], is a glycine rich protein with no detectable homology outside of Lepidoptera. It functions as an antimicrobial peptide against a range of bacteria, with greater specificity to Gram-negative bacteria, and appears to be commonly and widely expressed across a range of life stages and tissues, with significant increases in expression observed following exposure to bacteria [75, 76].

### Gene expansion of lepidopteran sugar and solute transporters

**Fig. 4:**
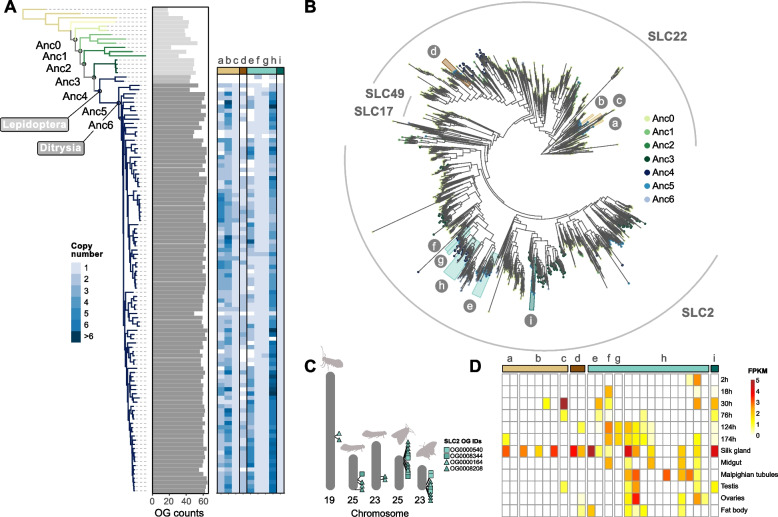
Origins and evolution of lepidopteran and ditrysian-specific sugar transporter genes. Orthogroups are identified as follows: a—OG0000700, b—OG0000319, c—OG0007512, d—OG0001801, e—OG0000540, f—OG0008208, g—OG0008344, h—OG0000164, i—OG000840 (**A**) Species tree on the left is coloured by taxonomic group, with the Lepidoptera and Ditrysia nodes labelled. The numbered ancestral nodes correlate to the node of origin for the orthogroups shown in the gene tree (part B). The grey bar chart (middle) shows that Lepidoptera (darker grey bars) have a higher total number of sugar transporter orthogroups compared to outgroup species. Copy number of lepidopteran and ditrysian-specific orthogroups varies greatly (heatmap, right): SLC22 transporters are below the light and dark brown bars (labelled a-d); SLC2 transporters are below the light and dark blue/green bars (labelled with letters e-i). **B** Phylogenetic tree built using a representative sample of outgroup sugar transporters, combined with sugar transporters identified in the Lepidoptera. SLC2 transporters are highlighted in light and dark blue/green along with letters e-i, while SLC22 transporters are highlighted in light and dark brown with letters a-d. Tip colours represent the node of origin (as shown in the species tree in part A) for each orthogroup. **C** The four closely related SLC2 transporters were mapped to a selection of lepidopteran chromosomes (left to right: Micropterix aruncella, Tinea trinotella, Tinea semifulvella, Papilio machaon and Autographa gamma). Sugar transporters of lepidopteran origin are represented by a triangle, while those of ditrysian origin are represented by a square. All four transporter orthogroups group in close physical proximity, on the same chromosome. **D** Heatmap of expression patterns of nine lepidopteran/ditrysian originating sugar transporters in Bombyx mori tissues

The orthogroup originating on the node leading to the Lepidoptera with the highest mean copy number is a sugar transporter gene family (OG0000164) (Fig. 3). Across the species analysed, the copy number for this lepidopteran-specific orthogroup ranged from one gene (*Micropterix aruncella*) to twelve genes (*Manduca sexta*). As the sugar transporter protein superfamily is large and diverse in animals [62], and to understand the significance of this Lepidoptera-specific orthogroup, we extended our analysis to include all orthogroups containing a sugar transporter domain. We found 99 orthogroups with genes possessing a sugar transporter domain present across all species in our dataset (Fig. 4A), nine of which are annotated as emerging on the lepidopteran or ditrysian node; gene copy number for all nine orthogoups in each species shows varying rates of copy number expansion and gene loss (Fig. 4A). Four of these orthogroups were single copy in all or most species, while five have undergone extensive gene duplication since their lepidopteran origins (Fig. 4A and B).

Next, we wanted to determine whether these nine lepidopteran sugar transporter-containing orthogroups had a single evolutionary origin or whether they had evolved independently from separate ancestral sugar transporter genes. To resolve this, a phylogenetic tree of transporter proteins from representative lepidopteran and outgroup species was constructed using all sugar transporter orthogroups (Fig. 4B). The tree topology suggests multiple origins of the lepidopteran- and ditrysian-specific sugar transporter genes, although not each of the nine orthogroups had independent origins. Notably, two lepidopteran-originating orthogroups (OG0000164 and OG0008208), and two ditrysian-originating orthogroups (OG0008344 and OG0000540) group close to each other in the phylogenetic tree (e–h). Another orthogroup (i; OG0008400) is located outside this clade, however each of these orthogroups are present in a larger clade consisting of members of the solute carrier 2 (SLC2) family (Fig. 4B). In some taxa SLC2 genes have been shown to encode proteins that facilitate transport of small sugars across cell membranes [62].

The remaining four sugar transporter orthogroups (a-d) are all ditrysian-specific, three of which form a single monophyletic group. The fourth orthogroup is located in a more phylogenetically distinct group, however, all orthogroups belong to the SLC22 protein subfamily (Fig. 4B). The SLC22 proteins are membrane transporters known to regulate metabolic functions, transporting a broader range of small molecules than SLC2 [62]. In all instances, the most closely related orthogroups in the gene tree contain both outgroups and lepidopteran species (Fig. 4B). This implies that the lepidopteran- and ditrysian-specific transporter orthogroups originated from more ancient gene families that were present across all or most insects including Lepidoptera. These ancestral genes duplicated and underwent extensive amino acid substitutions specifically in the lineage leading to Lepidoptera or Ditrysia.

The four SLC2-like orthogroups [e–h] which group closely together in the gene tree (Fig. 4B) are also co-located in the genome, found consistently in close association with one another across diverse lepidopteran species (Fig. 4C). This suggests that these sugar transporter genes originated from a single ancestral duplication event at the base of the Lepidoptera and subsequently underwent tandem duplication in the ancestral lepidopteran and again in the branch leading to Ditrysia. In contrast, for the SLC22-like orthogroups gained on the ditrysian branch (a-d), we do not see the same close linkage and instead they are scattered on separate chromosomes (Supplementary Figure S1). If these originated from a single ancestral gene, as suggested by branching patterns in the gene tree, they dispersed around the genome after duplication.

To investigate possible functions of these lepidopteran-species transporter gene families, we assessed their patterns of expression across multiple time points and tissues from *Bombyx mori* [65, 66]. All genes from the nine lepidopteran and ditrysian-specific gene families are expressed in at least one tissue or at one developmental time point (Fig. 4D). The silk gland was the most frequent site of expression across the nine orthogroups, but some of the genes have wide and distributed expression (Fig. 4D).

### Lepidoptera propellin genes arose through horizontal gene transfer

The second gene family which emerged at the base of Lepidoptera and is maintained in significantly high copy number across all butterfly and moth species analysed is orthogroup OG0000175 (Fig. 3). The genes in this family were previously undescribed in insects. Below we show they encode proteins with a beta-propeller structure; we therefore name this the *propellin* gene family. Intriguingly, this group of genes is conserved across Lepidoptera yet does not have detectable sequence identity to any orthogroups in the arthropod outgroups included in our initial analysis. Furthermore, iterative BLAST searching revealed that OG000175 is not the only set of *propellin* genes in Lepidoptera; the genes are split into eight distinct orthogroups, including the original group OG000175 with the highest copy number. All eight *propellin* orthogroups are specific to Lepidoptera (Supplementary Figure S2). Combining all *propellin* orthogroups together, we find Lepidoptera genomes have an average of 11 gene copies, ranging from 3 copies in *Neofacetella micropterix* (Micropterigidae) to 25 copies in *Phragmatobia fuliginosa* (Erebidae) (Supplementary Table S5).

To assess the likely origin of the *propellin* gene in Lepidoptera, we carried out a BLASTp search using all proteins in orthogroup OG0000175 against the NCBI nr protein database excluding Lepidoptera sequences. This revealed significant sequence similarity matches to proteins from bacterial species. The most frequent bacterial genus in the set of matches was *Spiroplasma*, with additional matches in *Macrococcus* and *Escherichia* (Supplementary Table S3). Using iterative rounds of BLAST searching, we found very few matches outside bacteria; we identified a potentially related unnamed gene in the genome of the plant *Picea sitchensis* (spruce; ABK22491.1) and a fungus gnat *Bradysia coprophila* (30% identity to a *Spiroplasma* homologue of Lepidoptera *propellin* genes over 16% query cover). To confirm the likely bacterial origins of the different *propellin* orthogroups, we also carried out BLASTp and tBLASTn searches against the nr and core nt databases, respectively, for each of the *propellin* orthogroups. We searched protein sequences from 10 representative species in each of the 8 orthogroups using both methods and found that bacterial, specifically *Spiroplasma*, sequences represented the majority of sequence similarity hits (Supplementary Table S4). While two genes from one orthogroup showed sequence similarity to other insect proteins (E3 ubiquitin ligases), we deduce that these hits are likely a result of spurious homology, with low query coverage (34–40%) and sequence similarity likely resulting from convergent amino acid residues in repetitive regions. In addition to this, all other hits from other species in the same orthogroup showed sequence similarity to *Spiroplasma* and other bacterial proteins. These *Spiroplasma* proteins were deduced to have similar tertiary structures to *propellin* (i.e. 6-bladed beta propeller; RMSD value of 3.73); in contrast, the spurious insect protein hits possessed multiple alpha helices and no structural similarity (RMSD value of 5.57).

**Fig. 5:**
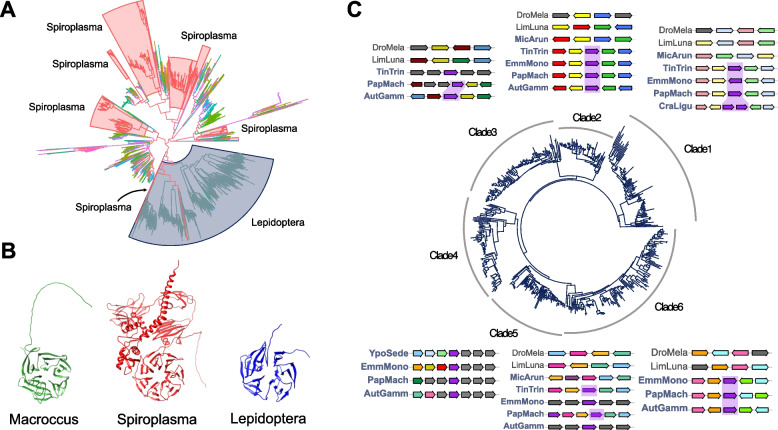
Lepidoptera-specific genes encoding proteins with sequence identity and structural similarity to bacterial 6-bladed propeller proteins. **A** Gene tree of propellin and putative bacterial homologs. The Lepidoptera clade (blue) and *Spiroplasma* clades (red) are labelled with coloured boxes and text. All other branches represent a range of bacterial species which are shown in Supplementary Figure S3. Molecular phylogenetic analysis indicates the propellin genes of Lepidoptera are monophyletic, whose most closely branching lineages are *Spiroplasma* genes, and sister group to a clade dominated by *Spiroplasma* genes (highlighted in red). **B** AlphaFold predictions suggest lepidopteran propellin proteins form 6-bladed propeller structures similar to bacterial homologues; examples shown from *Macrococcus* (green), *Spiroplasma* (red) and the lepidopteran *M. sexta* (blue). Additional protein structure predictions in Supplementary Figure S4. **C** Molecular phylogenetic analysis indicates that the largest orthogroup of lepidopteran propellin genes divides into 6 clades, each gene (purple) located at a different chromosomal location, most of which show conserved synteny between lepidopteran species (synteny indicated by shaded purple regions). The Micropterigidae species *M. aruncella* only has a gene in clades 1. Marker genes are shown by various colours

Next, we constructed a phylogenetic tree of all *propellin* copies and their putatively homologous sequences. This shows that all lepidopteran *propellin* sequences are closely related in the gene tree (Fig. 5A, Supplementary Figure S3). The most closely related branches to the Lepidoptera clade are *Spiroplasma* sequences which, along with a larger sister clade dominated by *Spiroplasma* sequences, suggests there has been a putative horizontal gene transfer from bacteria to Lepidoptera (Fig. 5A). Based on the gene tree topology, we cannot exclude the possibility of multiple horizontal transfer events into Lepidoptera. Although there are clear sequence similarity matches between Lepidoptera and *Spiroplasma*, the level of primary sequence identity is low. The highest percentage identity found between a lepidopteran protein and a *Spiroplasma* protein had only 35% identity over a sequence alignment of 134aa. This represents 45% coverage of a lepidopteran *propellin* protein (ENSAGMG00005008917.1) and 18% of a *Spiroplasma* protein (WP_164028422.1). To further assess legitimacy of the homologous relationships between these genes with low sequence identity, we predicted 3D structures of the deduced proteins from *Spiroplasma*, *Macrococcus*, and Lepidoptera (using six genes from *Manduca sexta* as representative of Lepidoptera) with AlphaFold [58] (Fig. 5B; Supplementary Figure S4). We find clear similarity in predicted protein structure with all lepidopteran and bacterial sequences having a 6-bladed propeller structure (Fig. 5B). Further support for homology between lepidopteran *propellin* proteins and bacterial proteins was found when we aligned representative protein structures, which showed an RMSD value of 3.83 and TM-score of 0.69 (Supplementary Figure S5). Each structured propeller region within a *propellin* protein is approximately 221aa long consisting of blades of 30aa in length. There is variability outside of the beta-propeller domain including regions of varying length and structure, most notably in the additional domains in the *Spiroplasma* protein model (Fig. 5B). To reflect this protein structure, we name the Lepidoptera genes the *propellin* gene family.

To further investigate the evolution of *propellin* genes, we focussed attention on the high-copy number *propellin* orthogroup (OG0000175; Supplementary Figure S2). Phylogenetic analysis divides this orthogroup into six clades within Lepidoptera, which we refer to as gene subfamilies (Fig. 5C). The early diverging lineage of Lepidoptera, represented by the family Micropterigidae, has a *propellin* gene in clade 1 in the gene tree (Fig. 5C). Next, we examined the local gene synteny for these six subfamilies across representative lepidopteran species. Genes from each subfamily, excluding clade four, were found in a microsyntenic cluster of genes (‘marker genes’) which are homologous across most or all species (Fig. 5C). This confirms that each of these 6 *propellin* subfamilies are one-to-one orthologues across Lepidoptera. Importantly, many of the marker genes also exist in microsyntenic blocks in the arthropod outgroups, consistent with these being the genomic sites where the Lepidoptera-specific *propellin* gene was integrated (Fig. 5C). Since the six *propellin* subfamilies are at distinct chromosomal locations, yet form a monophyletic group in molecular phylogenetic analysis, we propose that this orthogroup emerged through a single HGT event from *Spiroplasma* or another bacterial source to Lepidoptera, followed by duplication and transposition around the genome. These duplications generated not only the six subfamilies analysed in detail, but also likely the additional *propellin* genes referred to above. We note that genes in subfamily 1 are intronless (or have one intron), while the remaining subfamilies and additional *propellin* orthogroups have between 0 and 8 introns, with the median count being 1 intron. This could reflect transposition via an RNA intermediate or could be a legacy of the gene’s bacterial origin (Supplementary Table S5).

For a first insight into the possible functional role of the lepidopteran *propellin* genes, we analysed the expression of all copies of *propellin* using transcriptomic data sets from three species: *Bombyx mori*, *Danaus plexippus*, and *Papilio machaon* (Supplementary Figure S6). While we find evidence for expression of all gene copies in each species, the patterns are complex and variable within and between species. In *Danaus plexippus* for example, while most *propellin* copies show some expression in larval or pupal stages (8 of the 11 genes), levels of expression are highest in the adult life stage, with particularly high expression found in the thorax, compared to the head or abdomen (Supplementary Figure S6). In *Papilio machaon* most copies are restricted to one or two life stages, while others are strongly expressed throughout the life cycle of the butterfly. Expression in *Bombyx mori* shows wider coverage across life stages and tissue types, with most gene copies expressed in early developmental and adult life stages. While expression is common across most adult tissue types in *Bombyx mori*, there is little, or no expression found in the midgut or silk glands (Supplementary Figure S6). While there is little correlation in expression between homologous copies of *propellin* across all three species, we note that transcriptomic datasets available are not comprehensive. However, such pervasive expression across life stages and tissues in multiple species provides support to the fact that these genes are functional across a wide range of lepidopteran species.

We noted above that there were some sequence similarity matches outside bacteria and Lepidoptera. The putatively homologous gene from Diptera is an uncharacterised locus (LOC119081672, encoding putative protein XP_037046651) on an unplaced scaffold in the genome assembly of a fungus gnat *Bradysia coprophila* [77]. We find this gene is present in two species of *Bradysia.* It is unlikely that the fungus gnat scaffold is a contaminating sequence since it is present in two species, and because it is adjacent in the genomes to recognisable insect genes (Supplementary Figure S6). Analysis of the unplaced scaffold reveals clearly dipteran genes immediately 3’ (LOC119081668) and relatively close 5’ (LOC119081673 and LOC119081675) to the gene of interest. Intriguingly, a locus immediately 5’ (LOC119081585) has high similarity to springtail (*Collembola*) tyrosine kinases, and the next neighbouring gene (LOC119081673) is *Bradysia*-specific (Supplementary Figure S6). We therefore suggest the Diptera gene LOC119081672 arose by an independent HGT from *Spiroplasma* in the *Bradysia* fungus gnat genus, which has likely also acquired other genes by HGT. We have not deduced the origin of the loci with a sequence match in *Picea sitchensis* (spruce).

## Discussion

In this study we identified 217 ‘novel’ genes arising on the evolutionary lineage leading Lepidoptera, after it had diverged from outgroups including the closest related order Trichoptera (caddisflies). We caution, however, against this as a quantitative measure of genomic novelty. First, we are using a pragmatic definition of novelty that includes de novo genes, horizontally transferred genes, and gene duplication followed by sequence divergence; altering parameters relating to sequence divergence could increase or decrease the gene count [78]. To improve inference of new genes in early lepidopteran evolution, we employed a phylogenetically informed approach to construct gene families, minimising the effects of bias resulting from rapid sequence divergence [50]. Second, novelty at the Lepidoptera node could be ‘undercounted’ if some genome annotations are incomplete, particularly those of early diverging lepidopteran taxa. Third, there are factors that could spuriously ‘overcount’ novelty. For example, in our study around half the novel orthogroups were found sporadically in a small number of distantly related Lepidoptera species. This could indicate repeated gene loss following the origin of the novel gene but could also include ‘noise’ as a result of some proteins being grouped incorrectly due to spurious sequence identity. Secondary loss of genes from caddisfly genomes could theoretically cause overcounting of genes on the Lepidoptera node, but we have minimized this risk through use of four caddisfly genomes. We also noted a small degree of overcounting (< 2%) emerging from alternative genome annotation methods [79], but we accounted for this (see Methods). Specifically, the initial input data consisted of proteomes predicted from the Ensembl Genebuild annotation which incorporates RNA sequence data and filters poorly supported potential coding transcript proteins. A second method of genome annotation, the BRAKER method, is potentially less stringent and found some genes that had been missed by Genebuild. The difference amounted to just two orthogroups. The same caveats apply to counts of novel genes at other similarly deep phylogenetic nodes. Despite this caveat, we find it interesting that even more apparent gene novelty (541 gene families) dates to the node leading to Ditrysia. These genes require further analysis, but the observation suggests that the evolution of new biological traits continued during the early evolutionary radiation of moths. More important than an absolute number of novel genes, the analysis gives us a first look into the relative importance of different modes of gene origin during the emergence of Lepidoptera. We find the majority of novel gene families gained on the ancestral lepidopteran branch arose via gene duplication and divergence (~ 82%) while around ~ 18% genes had no sequence matches or any recognisable domains. These are putative candidates for genes arising de novo from non-coding genes. Just two genes (< 1%) are candidates for having arisen via HGT (including the *propellin* gene), with hits to bacterial or fungal species.

One of the genes that likely arose via HGT was highlighted in our analysis as a novel gene that underwent extensive gene duplication in Lepidoptera to generate a large gene family. This gene family, which we name the *propellin* genes, is potentially functional as evidenced by the extensive retention through evolution and conserved domain structure. Currently, however, its precise role in lepidopteran biology is unclear. Phylogenetic analyses suggest that the progenitor of the *propellin* gene family was transferred to an insect from *Spiroplasma* bacteria, some time on the Lepidoptera stem lineage. *Spiroplasma* is a well-known intracellular symbiont in arthropods. Furthermore, *Spiroplasma* is known to colonise reproductive tissues, which in turn impacts upon the host’s reproduction, and indeed this genus is one of two bacterial symbionts in Lepidoptera for which maternal transmission has been demonstrated [80]. In some cases, transmission is enhanced by manipulation of host physiology, such as male-killing which increases the number of female offspring as observed in *Danaus chrysippus* [81]. Clearly, persistent association with reproductive tissues gives opportunity for horizontal gene transfer, as the symbiont DNA is in close physical proximity to the DNA of the host germline. This has been seen in the relationship between a mealybug and two endosymbiont species *Tremblaya* and *Moranella* [82]. Interestingly, we also found a putatively homologous gene in two species of Diptera (genus *Bradysia*), possibly reflecting an independent HGT event. This is consistent with previous findings that some types of gene are more prone to HGT than others, perhaps those encoding proteins with few interaction partners [83]. The evolutionary retention of the likely HGT-derived *propellin* gene, plus its extensive gene duplication in Lepidoptera, suggest this gene family likely evolved to perform functions that are important for the biology of moths and butterflies. We do not know the biological role, or roles, of *propellin* genes in Lepidoptera, but note that their bacterial homologues have diverse functions including ligand-binding proteins, signalling proteins, lysases, structural proteins, isomerases and hydrolases [84]. It is worth noting that the *Spiroplasma* genes which group closest to the *propellin* genes in the gene tree are annotated as hypothetical proteins without known function, suggesting more work is needed to understand the functional context of this gene.

The only other novel lepidopteran gene to show such widespread retention and extensive gene duplication encodes a family of SLC2-like sugar transporter proteins. In other animals, members of the SLC2 sugar transporter superfamily encode glucose-uptake proteins, ribose transport proteins, and several putative membrane proteins probably involved in sugar transport [62, 85, 86]. The functional link to sugars is particularly intriguing since the ecological association between Lepidoptera and sugar-feeding changed markedly in early lepidopteran evolution. Specifically, adult moths in the basal family Micropterigidae primitively lack a proboscis and are pollen feeders, whereas adult moths and butterflies in the Ditrysia use a proboscis to access sugar-rich nectar in flowers. Our wider comparative survey of sugar transporter gene families picks up potentially interesting co-evolution between this ecological shift and the sugar transporter genes. We find that although OG0000164 (and one other sugar transporter gene family) are present in pollen-feeding Micropterigidae, it is not until the evolution of the nectar-feeding Ditrysia that we see extensive gene duplication, widespread gene retention and the emergence of additional SLC-like sugar transporter gene families [62]. We suggest, therefore, that novel sugar transporter gene families emerged at the base of Lepidoptera, but it was only later in lepidopteran evolution that massive gene duplication and functional divergence of sugar-transporter genes took place, in association with nectar feeding. The causal link between these genetic changes and the evolution of novel feeding behaviour in the early evolution of Lepidoptera warrants further study.

## Conclusion

We have demonstrated the emergence of 217 novel gene families (orthogroups) on the node leading to Lepidoptera and 541 novel gene families emerging on the node leading to the Ditrysia. Two orthogroups have significantly higher gene copy per species across Lepidoptera indicative of extensive gene duplication following their origins. One likely originated by horizontal gene transfer from the endosymbiont bacterium *Spiroplasma* and then duplicated to generate a diverse group of ‘*propellin*’ genes encoding a 6-bladed propeller domain. The other encodes a large set of sugar transporter proteins and is part of a diverse set of sugar and solute transporter genes that duplicated and diverged extensively in early lepidopteran evolution.

## Supplementary Information

Supplementary Material 1.

Supplementary Material 2.

## Data Availability

Genome data associated with this study is listed in Supplementary Table S1 along with accession numbers. Data and code generated in this study can be found on figshare; figshare.com/s/32d1b9055257dad1892f.
