# Supplementary material for: Gene novelty and gene family expansion in the early evolution of Lepidoptera
Source: BMC Genomics. 2025 Feb 19;26:161. doi: 10.1186/s12864-025-11338-x (PMC11837612; doi:10.1186/s12864-025-11338-x)
Supplement: Supplementary file 1 — Supplementary Material 1. [file 12864_2025_11338_MOESM1_ESM.pdf]

Supplementary Figures - Gene novelty and gene family expansion in the early evolution of Lepidoptera

Asia E. Hoile[1], Peter W.H. Holland[1,*], and Peter O. Mulhair[1,*]

[1]Department of Biology, University of Oxford, 11a Mansfield Road, Oxford OX1 3SZ, UK
[*]Corresponding authors: Peter W.H. Holland, peter.holland@biology.ox.ac.uk; Peter O. Mulhair, peter.mulhair@biology.ox.ac.uk

Supplementary Figures

Supplementary Figure S1: Genome locations of all sugar transporter genes in *Autographa gamma*.
Supplementary Figure S2: Presence and copy number of all propellin orthogroups.
Supplementary Figure S3: Gene tree of all propellin gene copies and all homologous sequences with significant BLAST hits.
Supplementary Figure S4: Protein models of all propellin gene copies from *Manduca sexta*, *Micropterix aruncella*, and *Neomicropterix facetellas*.
Supplemental Figure S5: Structural protein alignment of *Maduca sexta propellin* and Spiroplasma homolog.
Supplemental Figure S6: Expression profile of *propellin* genes in three species.
Supplementary Figure S7: Gene tracks showing location and synteny of putative horizontally tranfered genes in two Bradysia species.

Supplementary References

Reference list for genomes listed in Supplementary Table S1.

Supplementary Figures

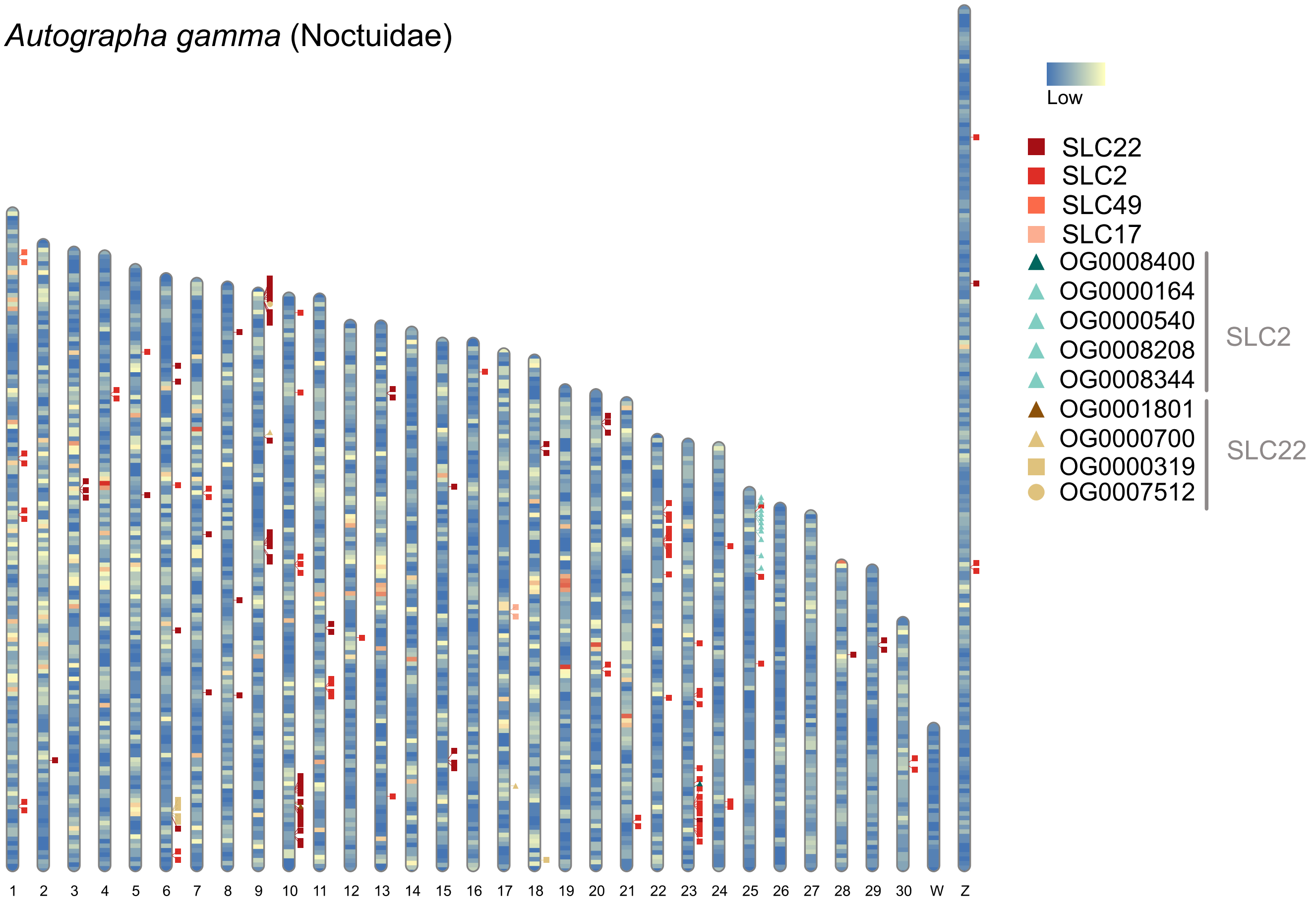

Supplemental Figure S1: Genome locations of all sugar transporter genes in *Autographa gamma*. Chromosome plot for the genome of *Autographa gamma*. Gene density across all chromosomes is represented on the chromosomes plots by a colour range of blue (low density) to red (high density). The location of all sugar transporter genes are annotated as coloured shaped along the chromosomes. The classification of sugar transporters gene types and the specific genes which underwent duplication within Lepidoptera are described in the figure legend to the right.

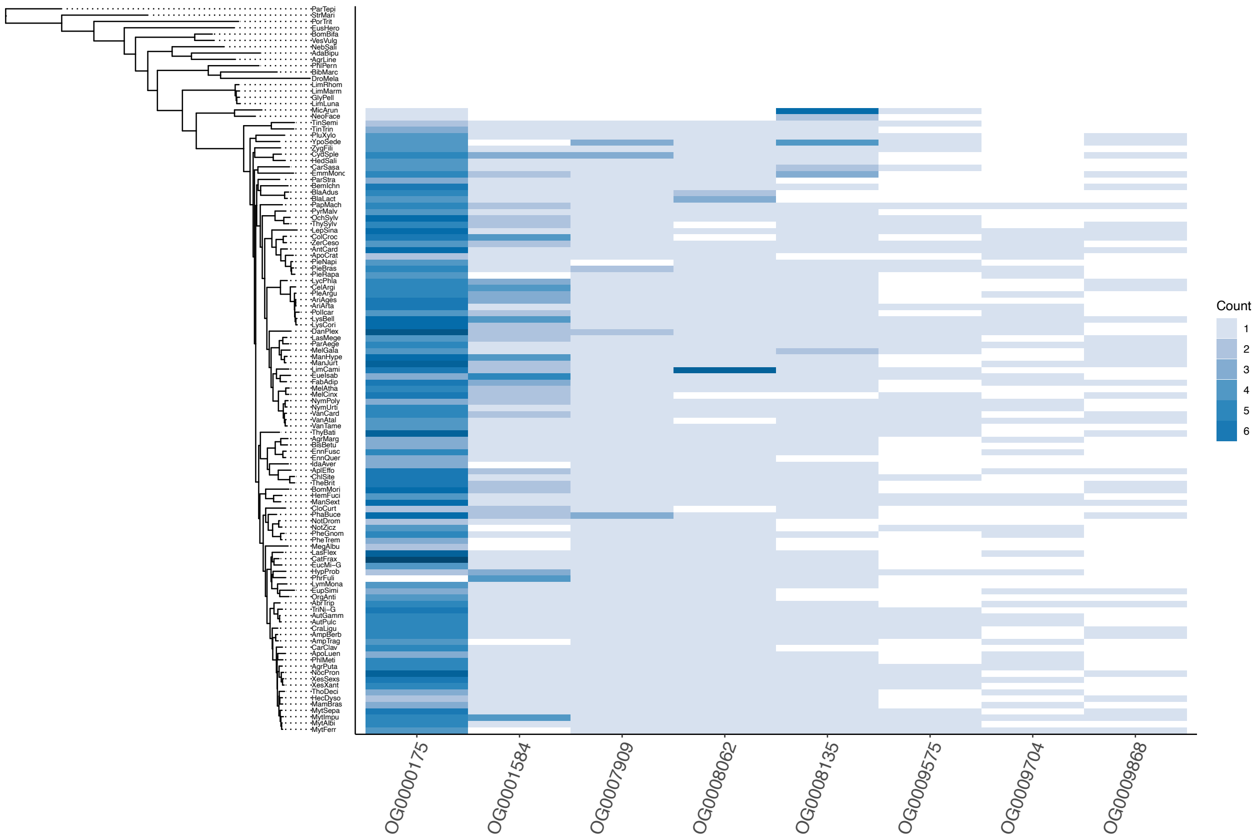

Supplemental Figure S2: Presence and copy number of all *propellin* orthogroups. Species tree of all species in the study is shown to the right. The presence and copy number of *propellin* genes are shown in the heatmap to the right. White indicates absence of the gene in a species, while presence and copy number are shown by varying shades of blue. The x axis represents each of the known *propellin* orthogroups in our dataset.

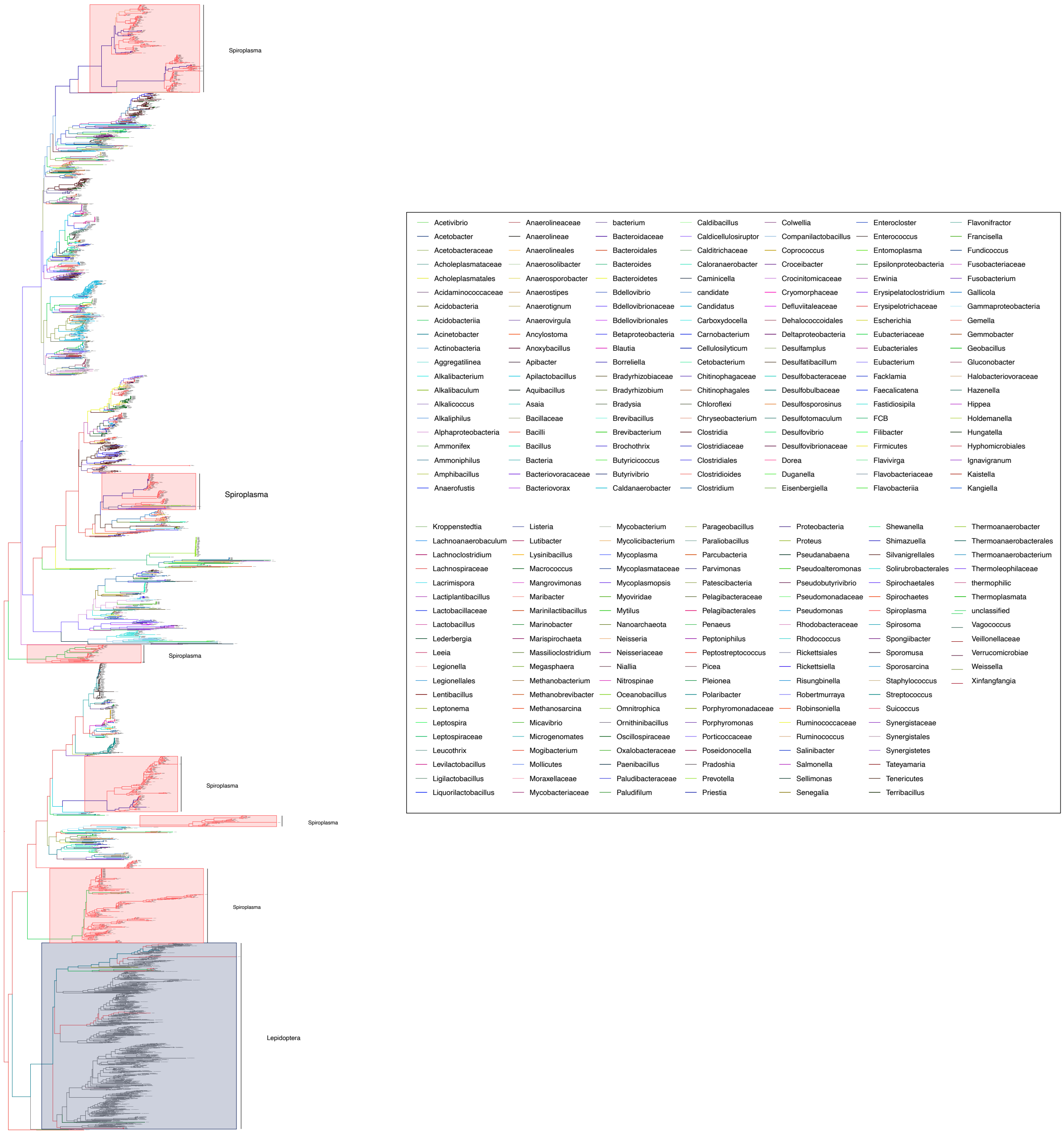

Supplemental Figure S3: Gene tree of all *propellin* gene copies and all homologous sequences with significant BLAST hits. Same gene tree as shown in Figure 5A in the main manuscript with tip names included. Tip names show species and ensembl gene IDs for the lepidopteran clade (Blue) and genus name for all homologous sequences, including Spiroplasma (red clades).

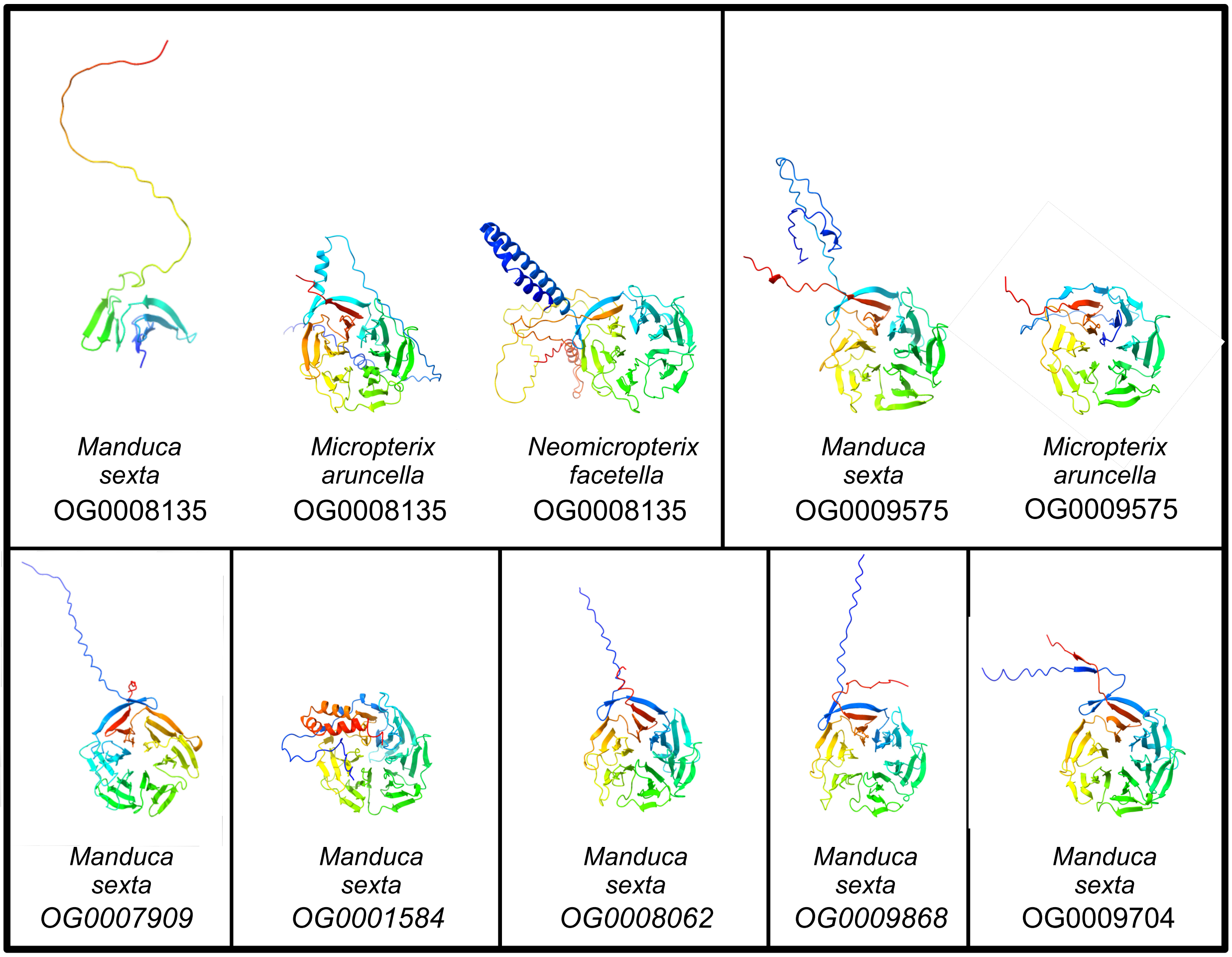

Supplemental Figure S4: Protein models of all *propellin* gene copies from *Manduca sexta*, *Micropterix aruncella*, and *Neomicropterix facetella*. Each of the propellin orthogroups are represented by protein models in three lepidopteran species. Protein models were inferred using AlphaFold, which show the 6 propeller blades along with additional, often disordered, structures within the protein. Each model is labelled with the orthogroup ID to which is belongs and the species from which it was inferred.

	Entry	Chain	RMSD	TM-score	Identity	Aligned Residues	Sequence Length	Modeled Residues
	ManSex_clade1_OG0000175_prediction.pdb	A	-	-	-	-	248	248
	Spiroplasma_QED25617_prediction.pdb	A	3.83	0.69	18%	181	830	830

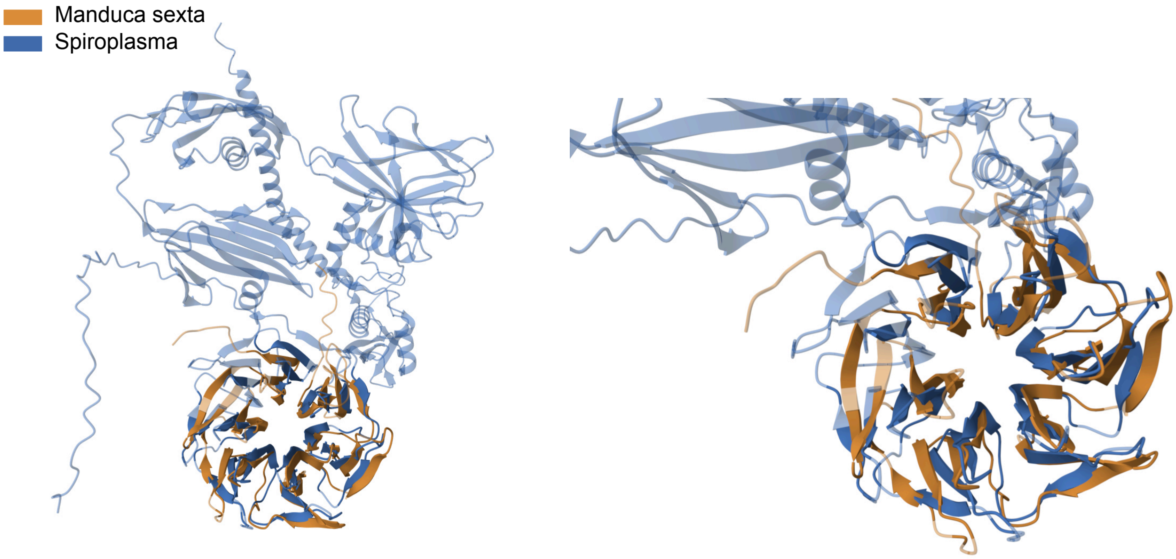

Supplemental Figure S5: Structural protein alignment of *Maduca sexta propellin* and Spiroplasma homolog. Structural alignment of *propellin* and a Spiroplasma homolog inferred using RCSB (REF). Table on top shows the RMSD value and TM-score when the Spiroplasma protein is aligned to the propellin protein. Images of protein structures below display this alignment, with Manduca sexta protein in orange and Spiroplasma protein in blue. The protein alignment on the right is zoomed in to give a closer view of the aligned regions corresponding to the full *propellin* sequence of *Manduca sexta*.

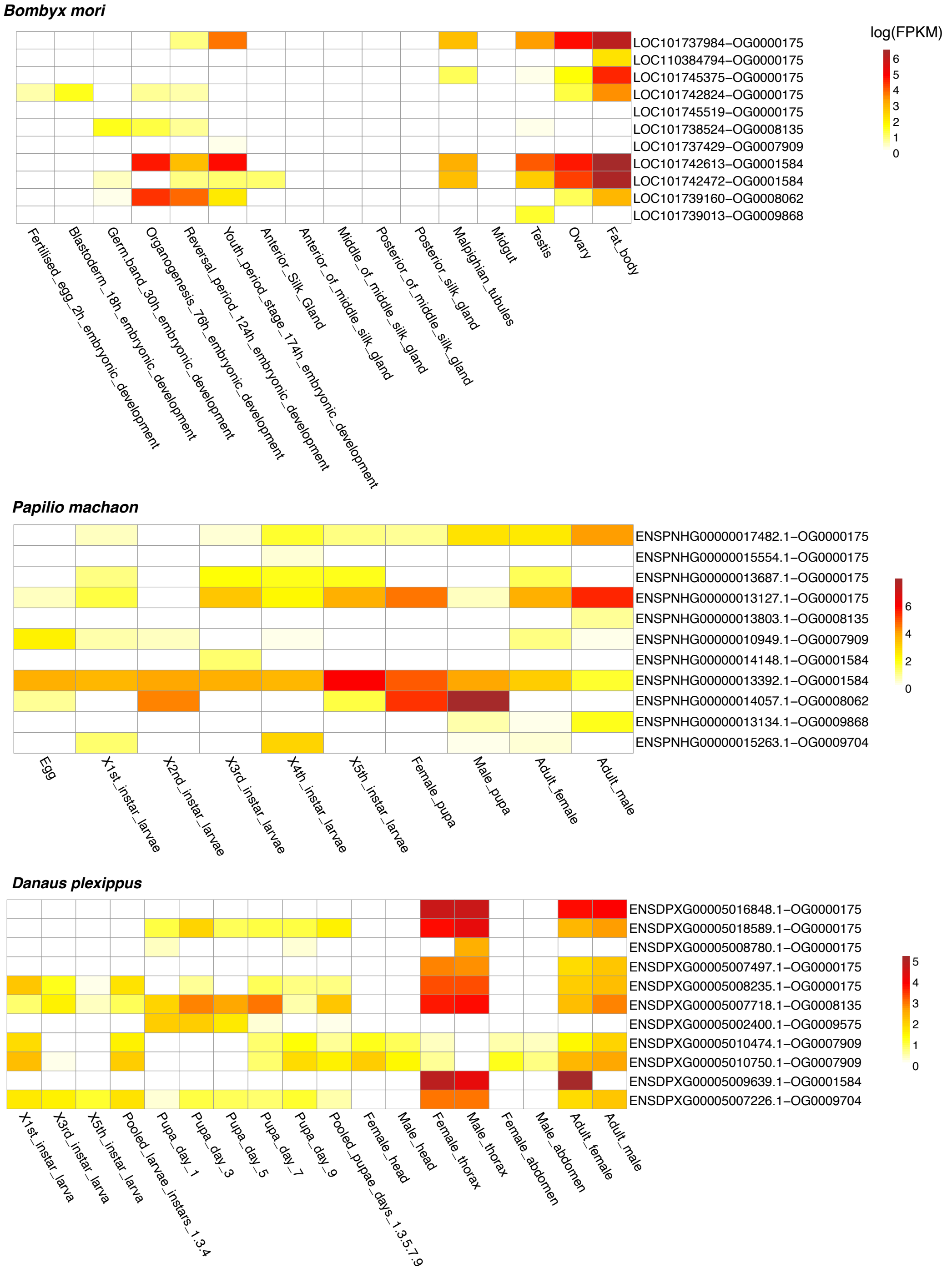

Supplemental Figure S6: Expression profile of *propellin* genes in three species. Left; Expression of all *propellin* gene copies in three lepidopteran species, *Bombyx mori*, *Papilio machaon*, and *Danaus plexippus*. Each plot shows expression in natural log transformed fpkm values, with the rows represented by different *propellin* gene copies and the columns representing a life stage or tissue type. The amount and type of RNA data differs between each species, but the columns are ordered from earlier life stages on the left to later stages and tissue types to the right. The orthogroup IDs to which the *propellin* copies belong to are annotated beside the gene IDs.

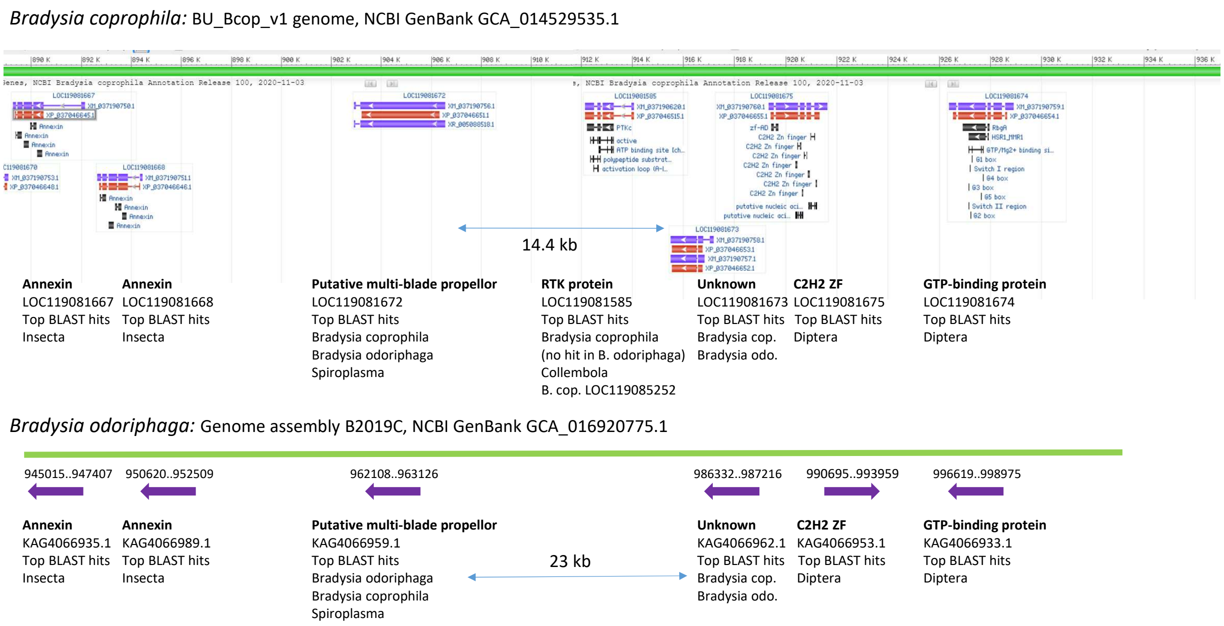

Supplemental Figure S7: Gene tracks showing location and synteny of putative horizontally tranfered genes in two Bradysia species. Gene location and synteny of potenital propellin homologs are shown for two fungus gnat species, *Bradysia coprophila* and *Bradysia odoriphaga*. The genome accession IDs for both species are give in the gene track titles and the potential horizontally transferred genes homologous to *propellin* are labelled as 'Putative multi-blade propellor'. Their orientation and surrounding genes are labeled with arrows and suggested gene names. Homology of the surrounding genes are given as the top BLAST hits using the ncbi BLAST search.

Supplementary References

Austin, M. et al., 2023. The genome sequence of a caddisfly, Limnephilus lunatus (Curtis, 1834). Wellcome Open Research , 8(25).

Baylor College of Medicine, 2011. Genome assembly $\mathrm{Smar}_1.0, s.l. : s.n.$

Beijing Academy of Agriculture and Forestry Sciences, 2020. Carposina sasakii genome , Beijing: s.n.

Bisschop, G. et al., 2021. The genome sequence of the small tortoiseshell butterfly, Aglais urticae (Linnaeus, 1758). Wellcome Open Research , 6(233).

Boyes, D., Broad, G., University of Oxford and Wytham Woods Genome Acquisition Lab, H. P. Natural History Museum Genome Acquisition Lab. 2023. The genome sequence of the Shuttle-shaped Dart, Agrotis puta (Hubner, 1803). Wellcome Open Research, 8(84).

Boyes, D. et al., 2022. The genome sequence of the ringed china-mark, Parapoynx stratiotata (Linnaeus, 1758). University of Oxford and Wytham Woods Genome Acquisition Lab, 7(121).

Boyes, D., Crowley, L., Holland, P. University of Oxford and Wytham Woods Genome Acquisition Lab., 2021. The genome sequence of the spectacle, Abrostola tripartita Hufnagel, 1766. Wellcome Open Research, 6(330).

Boyes, D., Crowley, L. H. P. University of Oxford and Wytham Woods Genome Acquisition Lab., 2021. The genome sequence of Svensson's copper underwing, Amphipyra berbera Rungs, 1949. Wellcome Open Research, 6(314).

Boyes, D. et al., 2022. The genome sequence of the 6-spot burnet, Zygaena filipendulae (Linnaeus, 1758). Wellcome Open Research , 7(197).

Boyes, D. et al., 2023. The genome sequence of the Beautiful Golden Y, Autographa pulchrina (Haworth, 1809). Wellcome Open Research, 8(375).

Boyes, D. et al., 2023. The genome sequence of the White-backed Marble, Hedya salicella (Linnaeus, 1758). Wellcome Open Research , 8(219).

Boyes, D. et al., 2023. The genome sequence of the Lesser Swallow Prominent, Pheosia gnoma (Fabricius, 1777). Wellcome Open Research , 8(192).

Boyes, D. Hammond, J., 2023. The genome sequence of the Marbled Piercer, Cydia splendana (Hübner, 1799). Wellcome Open Research, 8(436).

Boyes, D. Holland, P., 2022. The genome sequence of the silver Y moth, Autographa gamma (Linnaeus, 1758). Wellcome Open Trust, 7(100).

Boyes, D. et al., 2021. The genome sequence of the iron prominent, Notodonta dromedarius (Linnaeus, 1767). Wellcome Open Research, 6(341).

Boyes, D. et al., 2021. The genome sequence of the peach blossom moth, Thyatira batis (Linnaeus, 1758). Wellcome Open Research , 6(267).

Boyes, D. et al., 2021. The genome sequence of the snout, Hypena proboscidalis (Linnaeus, 1758). Wellcome Open Research , 6(236).

Boyes, D. et al., 2021. The genome sequence of the swallow prominent, Pheosia tremula (Clerck, 1759). Wellcome Open Research , 6(335).

Boyes, D. et al., 2021. The genome sequence of the yellow-tail moth, Euproctis similis (Fuessly, 1775). Wellcome Open Research , 6(227).

Boyes, D. et al., 2022. The genome sequence of the angle shades moth, Phlogophora meticulosa (Linnaeus, 1758). Wellcome Open Trust, 7(89).

Boyes, D. et al., 2022. The genome sequence of the buff-tip, Phalera bucephala (Linnaeus, 1758). Wellcome Open Research , 7(28).

Boyes, D. et al., 2022. The genome sequence of the Clifden nonpareil, Catocala fraxini (Linnaeus, 1758). Wellcome Open Research , 7(129).

Boyes, D. et al., 2022. The genome sequence of the large yellow underwing, Noctua pronuba (Linnaeus, 1758). Wellcome Open Research , 7(119).

Boyes, D. et al., 2022. The genome sequence of the square-spot rustic, Xestia xanthographa (Schiffermuller, 1775). Wellcome Open Research , 7(37).

Boyes, D. et al., 2023. The genome sequence of the Feathered Gothic, Tholera decimalis (Poda, 1761). Wellcome Open Research, 8(200).

Boyes, D. et al., 2023. The genome sequence of the Northern Deep-brown Dart, Aporophyla lueneburgensis (Freyer, 1848). Wellcome Open Research , 8(149).

Boyes, D. et al., 2023. The genome sequence of the Red-green Carpet, Chloroclysta siterata (Hufnagel, 1767). Wellcome Open Research, 8(206).

Boyes, D. et al., 2024. The genome sequence of the White-point, Mythimna albipuncta (Denis Schiffermüller, 1775). Wellcome Open Research , 9(62).

Boyes, D. et al., 2023. The genome sequence of the Dingy Dowd, Blastobasis adustella (Walsingham, 1894). Wellcome Open Research , 8(407).

Boyes, D. et al., 2023. The genome sequence of the London Dowd, Blastobasis lacticolella (Rebel, 1939). Wellcome Open Research, 8(352).

Boyes, D. et al., 2022. The genome sequence of the bird's nest moth, Tinea trinotella (Thunberg, 1794). Wellcome Open Research , 7(124).

Boyes, D. et al., 2022. The genome sequence of the black arches, Lymantria monacha (Linnaeus, 1758). Wellcome Open Research , 7(128).

Boyes, D. et al., 2022. The genome sequence of the clay, Mythimna ferrago (Fabricius, 1787). Wellcome Open Research , 7(177).

Boyes, D. et al., 2022. The genome sequence of the lesser treble-bar moth, Aplocera efformata (Guenee, 1857). Wellcome Open Research, 7(303).

Boyes, D. et al., 2022. The genome sequence of the pale mottled willow, Caradrina clavipalpis (Scopoli, 1763). Wellcome Open Research , 7(225).

Boyes, D. et al., 2022. The genome sequence of the pebble prominent, Notodonta ziczac (Linnaeus, 1758). Wellcome Open Research, 7(111).

Boyes, D. et al., 2022. The genome sequence of the smoky wainscot, Mythimna impura (Hubner, 1808). Wellcome Open Research , 7(226).

Boyes, D. et al., 2023. The genome sequence of the Coronet, Craniophora ligustri (Denis and Schiffermuller, 1775). Wellcome Open Research , 8(81).

Boyes, D. et al., 2023. The genome sequence of the Diamondback Moth, Plutella xylostella (Linnaeus, 1758). Wellcome Open Research , 8(404).

Boyes, D. et al., 2023. The genome sequence of the Fulvous Clothes Moth, Tinea semifulvella (Haworth, 1828). Wellcome Open Research , 8(104).

Boyes, D. et al., 2023. The genome sequence of the Mother Shipton moth, Euclidia mi (Clerck, 1759). Wellcome Open Research , 8(108).

Boyes, D. et al., 2023. The genome sequence of the Riband Wave, Idaea aversata (Linnaeus, 1758). Wellcome Open Research , 8(45).

Boyes, D. et al., 2023. The genome sequence of the Six-belted Clearwing, Bembecia ichneumoniformis (Denis Schiffermuller, 1775). Welcome Open Research, 8(515).

Boyes, D. et al., 2023. The genome sequence of the Spruce Carpet Moth, Thera britannica (Turner, 1925). Wellcome Open Research, 8(114).

Boyes, D. et al., 2023. The genome sequence of the Common Plume moth, Emmelina monodactyla (Linnaeus, 1758). Wellcome Open Research, 8(97).

Boyes, D. et al., 2023. The genome sequence of the Ruby Tiger, Phragmatobia fuliginosa (Linnaeus, 1758). Wellcome Open Research , 8(124).

Boyes, D. Mallick, T., 2023. The genome sequence of the Chocolate-tip, Clostera curtula (Linnaeus, 1758). Wellcome Open Research , 8(405).

Boyes, D. et al., 2023. The genome sequence of the Six-striped Rustic, Xestia sexstrigata (Haworth, 1809). Wellcome Open Research , 8(399).

Boyes, D. et al., 2023. The genome sequence of the August Thorn, Ennomos quercinarius (Hufnagel, 1767). Wellcome Open Research, 8(490).

Boyes, D. et al., 2023. The genome sequence of the Black-tipped Ermine, Yponomeuta plumbella (Denis Schiffermüller, 1775). Wellcome Open Research , 8(246).

Boyes, D. et al., 2022. The genome sequence of the Kent black arches, Meganola albula (Denis Schiffermuller, 1775). Wellcome Open Research , 7(310).

Boyes, D. University of Oxford and Wytham Woods Genome Acquisition Lab., 2023. The genome sequence of the Mouse Moth, Amphipyra tragopoginis (Clerck 1759). Wellcome Open Research, 8(54).

Boyes, D., Wright, C. University of Oxford and Wytham Woods Genome Acquisition Lab., 2022. The genome sequence of the peppered moth, Biston betularia Linnaeus, 1758. Wellcome Open Research, 7(97).

Broad, G. et al., 2023. The genome sequence of the Small Ranunculus, Hecatera dysodea (Denis Schiffermuller, 1775). Wellcome Open Research , 8(101).

Broad, G. et al., 2023. The genome sequence of a caddisfly, Limnephilus rhombicus (Linnaeus, 1758). Wellcome Open Research , 8(170).

Center for Ecological and Environmental Sciences, 2021. Genome assembly ASM1759143v1, s.l.: s.n.

Clifford, C. et al., 2023. The genome sequence of the cinnamon sedge caddisfly, Limnephilus marmoratus (Curtis, 1834). Wellcome Open Research , 8(64).

Crowley, L. et al., 2021. The genome sequence of the common wasp, Vespula vulgaris (Linnaeus, 1758). Wellcome Open Research , 6(232).

Ebdon, S. et al., 2022. The genome sequence of the orange-tip butterfly, Anthocharis cardamines (Linnaeus, 1758). Wellcome Open Research, 7(260).

Ebdon, S. et al., 2022. The genome sequence of the northern brown argus, Aricia artaxerxes (Fabricius, 1793). Wellcome Open Research , 7(314).

Ebdon, S. et al., 2021. The genome sequence of the clouded yellow, Colias crocea (Geoffroy, 1785). Wellcome Open Research, 6(284).

Ebdon, S. et al., 2022. The genome sequence of the black-veined white butterfly, Aporia crataegi (Linnaeus, 1758). Wellcome Open Research, 7(81).

Etxabe, A. et al., 2023. The genome sequence of the Cabbage Moth, Mamestra brassicae (Linnaeus, 1758). Wellcome Open Research , 8(486).

Genomes, I., 2020. Micropterix facetella GCA$_0$27564375.1$, Silver Spring: s.n.$

Graduate School of Agricultural and Life Sciences, The University of Tokyo, 2020. Bmori$_2$016v1.0$, Tokyo: GCF_0$14905235.1.

Hayward, A. et al., 2022. The genome sequence of the small skipper, Thymelicus sylvestris (Poda, 1761). Wellcome Open Research , 7(35).

Hayward, A. et al., 2022. The genome sequence of the silver-studded blue, Plebejus argus (Linnaeus, 1758). Wellcome Open Research , 7(315).

Hayward, A. et al., 2023. The genome sequence of the Brown Argus, Aricia agestis (Denis Schiffermüller, 1775). Wellcome Open Research, 8(336).

Hayward, A. et al., 2021. The genome sequence of the heath fritillary, Melitaea athalia (Rottemburg, 1775). Wellcome Open Research , 6(304).

Hayward, A. et al., 2022. The genome sequence of the grizzled skipper, Pyrgus malvae (Linnaeus, 1758). Wellcome Open Research , 7(114).

Hayward, A. et al., 2021. The genome sequence of the holly blue, Celastrina argiolus (Linnaeus, 1758). Wellcome Open Research, 6(340).

Holland, P. et al., 2023. The genome sequence of the White-barred Gold, Micropterix aruncella (Scopoli, 1763). Wellcome Open Research, 8(1).

Infravec, 2022. Genome assembly P.perniciosus$_a sm_v$2.0$, s.l.: s.n.$

John Hopkins University, 2020. Manduca sexta GCF$_0$14839805.1, $Baltimore : s.n.$

Lees, D., Boyes, D. Natural History Museum Genome Acquisition Lab., 2023. The genome sequence of the Dotted Border, Agriopis marginaria (Fabricius, 1776). Wellcome Open Research, 8(152).

Lohse, K. et al., 2021. The genome sequence of the small white, Pieris rapae (Linnaeus, 1758). Wellcome Open Research , 6(273).

Lohse, K. et al., 2021. The genome sequence of the red admiral, Vanessa atalanta (Linnaeus, 1758). Wellcome Open Research , 6(356).

Lohse, K. et al., 2022. The genome sequence of the common yellow swallowtail, Papilio machaon (Linnaeus, 1758). Wellcome Open Research , 7(261).

Lohse, K. et al., 2022. The genome sequence of the Adonis blue, Lysandra bellargus (Rottemburg, 1775). Wellcome Open Research , 7(255).

Lohse, K. et al., 2023. The genome sequence of the Large Skipper, Ochlodes sylvanus, (Esper, 1777). Wellcome Open Research, 8(75).

Lohse, K. et al., 2021. The genome sequences of the male and female green-veined white, Pieris napi (Linnaeus, 1758). Wellcome Open Research , 6(288).

Lohse, K. et al., 2022. The genome sequence of the wood white butterfly, Leptidea sinapis (Linnaeus, 1758). Wellcome Open Research , 7(254).

Lohse, K. et al., 2021. The genome sequence of the large tortoiseshell, Nymphalis polychloros (Linnaeus, 1758). Wellcome Open Research , 6(238).

Lohse, K. et al., 2021. The genome sequence of the small copper, Lycaena phlaeas (Linnaeus, 1760). Wellcome Open Research , 6(294).

Lohse, K. et al., 2021. The genome sequence of the large white, Pieris brassicae (Linnaeus, 1758). Wellcome Open Research , 6(262).

Lohse, K. et al., 2022. The genome sequence of the wall brown, Lasiommata megera (Linnaeus, 1767). Wellcome Open Research , 7(230).

Lohse, K. et al., 2022. The genome sequence of the wall brown, Lasiommata megera (Linnaeus, 1767). Wellcome Open Research , 7(230).

Lohse, K. et al., 2023. The genome sequence of the Common Blue, Polyommatus icarus (Rottemburg, 1775). Wellcome Open Research , 8(72).

Lohse, K. et al., 2021. The genome sequence of the speckled wood butterfly, Pararge aegeria (Linnaeus, 1758). Wellcome Open Research , 6(287).

Lohse, K. et al., 2022. The genome sequence of the high brown fritillary, Fabriciana adippe (Dennis Schiffermüller, 1775). Wellcome Open Research , 7 (298).

Lohse, K. et al., 2021. The genome sequence of the meadow brown, Maniola jurtina (Linnaeus, 1758). Wellcome Open Research , 6(296).

Lohse, K. et al., 2021. The genome sequence of the painted lady, Vanessa cardui Linnaeus 1758. Wellcome Open Trust , 6(324).

McSwan, E. et al., 2023. The genome sequence of the Mottled Sedge, Glyphotaelius pellucidus (Retzius, 1783). Wellcome Open Research , 8(102).

Mead, D. et al., 2021. The genome sequence of the ringlet, Aphantopus hyperantus Linnaeus 1758. Wellcome Open Research , 6(165).

Princeton University, 2019. Danaus plexippus plexippus GCA$_0$09731565.1, $Princeton : s.n.$

Rodriguez-Caro, L. et al., 2020. Genome Assembly of the Dogface Butterfly Zerene cesonia. Wellcome Open Research , 12(1), pp. 3580-3585.

ROTHAMSTEAD RESEARCH, 2022. Genome assembly PGI$_AGRIOTES_LIN_V1, s.l. : s.n.$

Sivell, O. et al., 2021. The genome sequence of the St Mark's fly, Bibio marci (Linnaeus, 1758). Wellcome Open Research , 6(285).

Sivell, O. et al., 2023. The genome sequence of a carabid beetle, Nebria salina (Fairmaire Laboulbène, 1854). Wellcome Open Research , 8(247).

The FlyBase Consortium; Berkeley Drosophila Genome Project ; Celera Genomics, 2014. Drosophila melanogaster $GCF_000001215.4, s.l. : s.n.$

The genome sequence of the Dusky Thorn, E. f. (. 1., 2023. Boyes, D.; Phillips, D.;. Wellcome Open Research, 8(505).

The i5k Initiative, 2019. Genome assembly $Ptep_3.0, s.l. : s.n.$

University of Alabama, 2020. Genome assembly $Bbif_JDL3187, Alabama : s.n.$

University of Bristol , 2021. Eueides isabella $GCA_019049475.1, Bristol : s.n.$

University of Exeter, 2018. Genome assembly $E_heros_v1.0_sep_2018, Exeter : s.n.$

University of Massachusetts Medical School, 2018. Trichoplusia ni (cabbage looper) $GCA_003590095.1, Massachusetts : s.n.$

USDA-ARS, 2018. Vanessa tameamea $GCA_002938995.1, s.l. : s.n.$

Vila, R.; Lohse, K.; Hayward, A.; Latesch, D.; Natural History Museum Genome Acquisition Lab; Darwin Tree of Life Barcoding collective; Wellcome Sanger Institute Tree of Life programme;, 2022. The genome sequence of the marbled white butterfly, Melanargia galathea (Linnaeus, 1758). Wellcome Open Research, 7(123).

Vila, R. et al., 2021. The genome sequence of the Glanville fritillary, Melitaea cinxia (Linnaeus, 1758). Wellcome Open Research , 6(266).

Vila, R. et al., 2023. The genome sequence of the Chalkhill Blue, Lysandra coridon (Poda, 1761). Wellcome Open Research , 8(162).

Vila, R. et al., 2022. The genome sequence of the white admiral, Limenitis camilla (Linnaeus, 1764). Wellcome Open Research , 7(301).

Weir, J. et al., 2023. The genome sequence of the Vapourer moth, Orgyia antiqua (Linnaeus, 1758). Wellcome Open Research, 8(314).

Wellcome Sanger Institute Tree of Life programme, et al., 2022. The genome sequence of the two-spot ladybird, Adalia bipunctata (Linnaeus, 1758). Wellcome Open Research, 7(288).

Wellcome Sanger Institute, 2021. Hemaris fuciformis $GCA_907164795.1, Cambridge : s.n.$

Yokoi, K. et al., 2022. Reference Genome Sequences of the Oriental Armyworm, Mythimna separata (Lepidoptera: Noctuidae). Insects, 13(12), p. 1172.
